# Stronger together: Coping behaviours and mental health changes of Canadian adolescents in early phases of the COVID-19 pandemic

**DOI:** 10.1186/s12889-023-15249-y

**Published:** 2023-02-13

**Authors:** Negin A. Riazi, Katelyn Battista, Markus J. Duncan, Terrance J. Wade, William Pickett, Mark A. Ferro, Scott T. Leatherdale, Karen A. Patte

**Affiliations:** 1 Department of Health Sciences, Brock University, 1812 Sir Isaac Brock Way, Saint Catharines, ON L2S 3A1 Canada; 2 School of Public Health Sciences, University of Waterloo, 200 University Ave West, Waterloo, ON N2L 3G1 Canada

**Keywords:** Coping, Youth, Adolescent, Mental health, COVID-19, Pandemic

## Abstract

**Background:**

The impact of the COVID-19 pandemic and consequent public health restrictions on the mental health of adolescents is of global concern. The purpose of this study was to examine how Canadian adolescents coped during the early pandemic and whether different coping methods were associated with changes in mental health from before the pandemic to the early lockdown response.

**Methods:**

Using two-year linked survey data (2018–2020) from a prospective cohort of secondary school students (n = 3,577), linear regression models were used to examine whether changes in mental health (anxiety [Generalized Anxiety Disorder-7 scale], depression [Center for Epidemiologic Studies Depression 10-item scale Revised], emotion regulation [Difficulties in Emotion Regulation Scale], psychosocial well-being [Flourishing scale]) were related to each coping behaviour.

**Results:**

The most common reported coping behaviours included staying connected with friends online (78.8%), playing video games, watching TV/movies, and/or surfing the internet/social media (76.2%), studying or working on schoolwork (71.0%), and getting exercise (65.2%). The use of positive coping mechanisms during the early pandemic period (e.g., keeping a regular schedule, time with family, time with friends online) was associated with less adverse mental health changes from before to during the early lockdown; whereas, negative coping mechanisms (e.g., spending time alone, eating junk food) were consistently associated with more adverse mental health changes.

**Conclusion:**

This study demonstrates the importance of social support and connections with both friends and family, as well as keeping and maintaining a routine, over the pandemic. Interventions supporting positive relationships and engagement in these coping behaviours may be protective for adolescent mental health during disruptive events.

**Supplementary Information:**

The online version contains supplementary material available at 10.1186/s12889-023-15249-y.

## Background

The World Health Organization declared the outbreak of the coronavirus disease a pandemic on March 11, 2020 [1]. In order to curb the spread of the virus, globally, countries instituted restrictions and lockdowns, including closures of physical locations (e.g., parks, schools) and non-essential workplaces, travel restrictions, bans on social gatherings, and the cancellation of extracurricular activities and many community programs and services [2, 3]. While these measures were necessary to slow the spread of the virus, they disrupted routine and learning, interrupted many primary prevention and health promotion strategies, and impeded social support [4].

Many pandemic-related changes are at odds with adolescents’ developmental propensity to seek autonomy from parents and parental figures and prioritize social connections. The resulting home isolation, limited opportunities to socialize with peers, cancellations of milestone events, and uncertainty about the future may have impacted the mental health and development of adolescents [5–7]. Globally, mental illness is the leading cause of disability among young people [8, 9], with the COVID-19 pandemic appearing to have exacerbated these concerns [10, 11]. Even before the pandemic, adolescents were concerned about their mental health [12]. A recent report by Children First Canada declared poor mental health as one of the top ten threats to childhood in Canada [13]. Kids Help Phone in Canada reported a 137% increase in the number of connections between 2019 (1.6 million) and 2020 (4.6 million) [13, 14].

The pandemic response not only introduced new stressors (i.e. any event, force, or condition that results in physical or emotional stress)[15], but may have interrupted many behaviours that adolescents had typically used to cope (e.g., socializing with friends, organized sports, etc.). A 2020 rapid review on the psychological impact of quarantine found that stressors included longer quarantine duration, fears associated with infection, frustration, and inadequate information [16]. Coping depicts a process of adaptation that is fundamentally important for long term functioning and development [17]. However, the need to constantly adapt both cognitive and behavioural efforts to address changing stressors may exceed a person’s capacity or resources to deal with those stressors [18]. Individuals cope with stress in different ways; their responses vary physiologically, psychologically, socially, and culturally[17]. Coping strategies have been categorized as either adaptive, such as problem-solving, support-seeking, and reappraisal, or maladaptive, such as avoidance, helplessness, isolation, and anxiety amplification[17, 19]. Exploring how adolescents were coping during the lockdown and what coping behaviours positively or negatively influenced changes in mental health is essential for understanding the impacts of the pandemic and to better inform appropriate intervention strategies.

The aim of the study was to examine coping behaviours during the early phases of the COVID-19 pandemic response in Canadian adolescents. Using two-year linked data from a prospective cohort of secondary school students, this study focused on two main questions: (1) How did adolescents cope during the early pandemic response? and (2) Are different coping methods differentially associated with changes in mental health from before the pandemic to the early lockdown response?

## Methods

### Design and participants

Student-level data from the Cannabis, Obesity, Mental health, Physical activity, Alcohol, Smoking, and Sedentary behaviour (COMPASS) study were used for analyses. The COMPASS study (2012–2027) collects hierarchical and longitudinal health survey data annually from a rolling cohort of students in Grades 9–12/Secondary 1–5 attending a convenience sample of secondary schools in the provinces of British Columbia (BC), Ontario (ON), Quebec (QC) and Alberta (AB). All students attending participating schools were invited to participate using active-information passive-consent parental permission protocols, which are critical for collecting robust data among adolescents [20, 21]. The student questionnaire, available in English and French, includes items to create a unique code for each respondent allowing COMPASS researchers to link each student’s data across years of participation (see [22]). Additional details regarding COMPASS study methods can be found online (www.compass.uwaterloo.ca) or in print [23]. Ethics approval for COMPASS has been obtained from the University of Waterloo (ORE#30,118), Brock University (REB#18–099), CIUSSS de la Capitale-Nationale–Université Laval (#MP-13-2017-1264), and participating school boards.

We used 2-year linked survey data collected from students that attended 49 secondary schools in ON (N = 20) and QC (N = 27), and BC (N = 2) that participated in the spring of the 2018/19 school year (Time 1; 84.2% response rate overall) and in May-July 2020 (Time 2; 29.2% response rate). In 2018/19, student data were collected using a paper-based survey completed during one classroom period by whole school samples. In 2020, as schools were closed for *in-person* learning due to the COVID-19 pandemic response, surveys were conducted online. A survey link was emailed to all students by their schools, followed by a reminder email one week after [24]. The majority of initial emails were sent out on May 12, 2020; the earliest school email was sent May 1, 2020 [24]. ON and BC surveys were left open for two weeks after the initial email, and QC surveys for 4 weeks. The last survey closed on July 6, 2020 [24]. The total linked sample from the 2018/19 to the 2020 online questionnaire included 4,113 students. However, 536 had missing data on mental health, covariates, or coping behaviours and were excluded. A complete case analysis was used as many students with missing data stopped completing the online survey (78.2% of the 536 cases were missing both outcome and coping behaviour response). Males and students in Grade 12 had higher rates of missing data on mental health outcome and COVID-19 coping behaviour than females and those in younger grades, though the magnitudes of these differences were small. Participants excluded due to missing values were similar to included participants in terms of weekly spending money and household size. The total available sample to undergo analyses was 3,577.

### Mental health outcomes

#### Depressive symptoms

Students self-reported depressive symptoms on the 10-item Center for Epidemiologic Studies Depression scale Revised (CESD10) [25]. Items asked for the frequency of depressive symptoms experienced within the last 7 days: “None or less than 1 day”, “1–2 days”, “3–4 days”, or “5–7 days.” Responses were scored from 0 to 3, respectively, and summed. Higher sum scores indicate greater depressive symptoms. The scale has demonstrated validity in adolescents [26–28] including measurement validity in the COMPASS sample [29]. The internal consistency in Time 1 was α = 0.83.

#### Anxiety symptoms

Students self-reported anxiety symptoms using the 7-item Generalized Anxiety Disorder scale (GAD7) [30]. Items asked students to indicate how frequently they experienced each symptom of anxiety in the last 2 weeks: “not at all”, “several days”, “over half the days”, or “nearly every day”. Responses were scored 0–3 respectively and summed. Greater anxiety experienced was indicated by higher sum scores. GAD7 scores have been shown to correlate strongly with other measures of anxiety in both clinical and non-clinical populations of adolescents [30–33], and measurement invariance in the current sample [29]. The internal consistency in Time 1 was α = 0.89.

#### Psychosocial well-being

Students’ psychosocial well-being was assessed using the 8-item Flourishing Scale (FS) [34, 35]. Items indicate feelings of competence, optimism, purpose, and success in personal relationships. The COMPASS student questionnaire [36, 37] uses a 5-point Likert-like scale ranging from “strongly disagree” to “strongly agree” (scored 1–5). Higher total scores reflect better psychosocial well-being. The FS has demonstrated validity in adolescent populations [38, 39] including measurement invariance for gender, grade, and ethnicity in the COMPASS study [39]. The internal consistency in Time 1 was α = 0.90.

#### Emotion regulation

Emotion regulation, or emotional skills, was assessed using a modified version of the Difficulties in Emotion Regulation Scale (DERS) [40]. For brevity in the COMPASS student questionnaire, the DERS was modified to include one item from each of the 6 subscales based on the highest factor loading from previous psychometric work among adolescents [40, 41]. For each item, students were asked to indicate how often the statement applies to them using a 5-point Likert scale from “Almost never” to “Almost always”. Items were summed to create a total DERS score, with higher scores reflecting greater emotion dysregulation. Recent analyses support measurement invariance and a one-factor solution in COMPASS sample (Romano et al., in preparation). The internal consistency in Time 1 was α = 0.68.

### Coping behaviours

Adapted from other COVID-19 related coping measures [42–44], coping behaviours were only measured at Time 2, when students were asked “How have you been coping with changes related to COVID-19?” and to select all that applied from a list of 20 items. Coping behaviour responses were entered individually in the models and categorized *post hoc* as follows: *Social behaviours* included staying connected with friends online, meeting up with my friends outside, and/or spending time with my family (e.g., playing games, eating meals together, hanging out). *Substance Use behaviours* included using cannabis/marijuana, drinking alcohol, smoking cigarettes, and/or vaping. *Emotional Eating behaviours* included eating junk food. *Passive Screen Activities behaviours* included playing video games, watching TV or movies, and/or surfing the internet/social media. Behaviours categorized as *Hobbies & the Arts* included reading, writing, playing music, or working on arts and crafts; learning something new (painting, playing a musical instrument, a new language); cooking/baking; and/or spending time with my dog/cat or other pet. Behaviours categorized as *Caring for others* and *Spirituality and mindfulness* included trying to help others and meditating/praying, respectively. *Structured Lifestyle behaviours* included getting exercise (e.g., getting outside to go for a walk or bike ride, working out at home), keeping to a regular schedule (e.g., waking up, eating meals, and going to bed around the same time as usual), and/or studying or working on schoolwork. *Formal Support* behaviours included connecting with mental health professionals and *Time Alone* behaviours encompassed spending time alone.

### Covariates

Sociodemographic characteristics to be included as covariates were first chosen based on known associations with mental health and coping behaviours, and next by generalized linear mixed models, first examining associations with changes in each mental health outcome and then examining which remaining factors were associated with each coping behaviour. Final models controlled for Time 2 measures of grade (Sect. 1, Sect. 2, Sect. 3/Gr 9, Sect. 4/Gr 10, Sect. 5/Gr 11, Gr 12, Other), gender (Female, Male, Describe Differently/Prefer not to say), province (BC, ON, QC), weekly spending money ($0, $1–20, $21–40, $41–100, More than $100, Don’t Know), and household size (1–2 people, 3, people, 4 people, 5 people, 6 or more people). Student race/ethnicity and school area urbanicity and median income were not included in final models as they did not demonstrate significant associations with changes in the mental health outcomes. Additionally, baseline mental health outcomes scores were included as a confounding predictor of change scores [45, 46].

### Analysis

Consistent with recommendations for Likert-type scales [47, 48], mental health scales were person-mean imputed for students missing 1 or 2 items and were set to missing for students missing 3 or more items. Missing data were imputed for 9.3% (7.3% missing 1 item) of participants for the CESD10, 4.6% (4.0% missing 1 item) for the GAD-7, 3.2% (2.7% missing 1 item) for the FS, and 3.16% (2.7% missing 1 item) for the DERS; sample distributions were similar before and after imputation. SAS was used to generate linear regression models to examine whether the change in mental health score (Time 2-Time 1) was related to each coping behaviour. Final models for each mental health outcome regressed change scores on all covariates and coping behaviours simultaneously. Gender by coping behaviour interactions were tested when a main effect was present for the individual coping behaviour. Interaction results for “I describe my gender differently/prefer not to say” were not reported due to small sample size. Low Intraclass Correlations (ICC) (all ICCs ≤ 1.65) for variability in mental health change scores did not indicate a need to control for school clustering. Correlational analyses did not indicate multicollinearity concerns (VIF < 2 in all cases).

## Results

### Descriptive statistics

See Table 1 for sample demographics based on student responses at Time 2 and change in mental health scores (Time 2-Time 1) among adolescents before (Time 1 [2019]) and during the early COVID-19 lockdown (Time 2 [2020]). Most students identified as female (64.2%) and were from QC (60.4%). Table 2 presents the behaviours students reported engaging in to cope with COVID-19 during the early lockdown and the model predicted parameter estimates for changes in depression, anxiety, psychosocial well-being, and emotion regulation scores based on whether students were engaging or not engaging in each coping behaviour.

**Table 1:** Demographic Characteristics at Time 2 (2020) and mental health scores among adolescents before (Time 1 [2019]) and during the early COVID-19 lockdown (Time 2 [2020]) (n = 3577)

Demographics at Time 2 (2020)		n	%
Grade	Section 1 (QC only)	21	0.6%
	Section 2 (QC only)	631	17.6%
	Section 3/Gr 9	623	17.4%
	Section 4/Gr 10	957	26.8%
	Section 5/Gr 11	939	26.3%
	Gr 12	375	10.5%
	Other	31	0.9%
Gender	Female	2297	64.2%
	Male	1247	34.9%
	Describe Differently/ Prefer not to say	33	0.9%
Province	BC	176	4.9%
	ON	1241	34.7%
	QC	2160	60.4%
Weekly Spending Money	Zero	692	19.3%
	$1-$20	631	17.6%
	$21-$40	300	8.4%
	$41-$100	358	10.0%
	More than $100	764	21.4%
	Don’t Know	832	23.3%
Household Size	1 or 2 people	223	6.2%
	3 people	690	19.3%
	4 people	1435	40.1%
	5 people	804	22.5%
	6 or more people	425	11.9%
**Mental Health Outcomes**	T1 (2019) M (SD)	T2 (2020) M (SD)	T2-T1 M (SD)
Depression (CESD10)	8.4 (6.0)	9.1 (6.2)	0.8 (5.5)
Anxiety (GAD7)	5.9 (5.2)	6.4 (5.6)	0.5 (4.7)
Psychosocial well-being (FS)	32.8 (5.5)	32.6 (5.5)	-0.2 (4.7)
Emotional dysregulation (DERS)	13.9 (4.7)	14.5 (4.7)	0.6 (4.5)

**Table 2:** Coping Behaviours (Time 2) and Parameter Estimates for Change in Depression, Anxiety, Psychosocial Well-being, and Emotion Regulation among adolescents from before (2019) to early COVID-19 lockdown (2020) by coping behaviours

Coping Behaviours at Time 2 (2020)	Parameter Estimate (Reference: Did Not Engage)																							
	Depression				Anxiety						Psychosocial Well-being						Emotional Dysregulation							
	(n = 3492)				(n = 3531)						(n = 3556)						(n = 3509)							
	**n (%)**	**b**	**95% CI**	**p-value**	**b**	**95% CI**			**p-value**		**b**		**95% CI**		**p-value**		**b**	**95% CI**			**p-value**			
**Social**																								
Staying connected with friends online	2817 (78.8)	-0.34	[-0.73, 0.05]	0.086	-0.24	[-0.6, 0.11]			0.174		1.22		[0.89, 1.55]		**< 0.0001**		-0.45	[-0.77, -0.13]			**0.006**			
Meeting up with friends outside	1198 (33.5)	-0.29	[-0.63, 0.05]	0.100	-0.15	[-0.45, 0.16]			0.346		0.54		[0.25, 0.84]		**0.000**		0.03	[-0.26, 0.31]			0.856			
Spending time with family	2095 (58.6)	-1.95	[-2.29, -1.60]	**< 0.0001**	-1.22	[-1.53, -0.91]			**< 0.0001**		1.52		[1.22, 1.81]		**< 0.0001**		-1.09	[-1.38, -0.81]			**< 0.0001**			
**Substance Use**																								
Cannabis	157 (4.4)	-0.31	[-1.18, 0.57]	0.490	-1.02	[-1.81, -0.23]			**0.012**		0.15		[-0.59, 0.89]		0.691		-0.06	[-0.78, 0.66]			0.863			
Alcohol	321 (9.0)	1.01	[0.40, 1.62]	**0.001**	0.81	[0.26, 1.36]			**0.004**		-0.71		[-1.23, -0.20]		**0.007**		0.38	[-0.13, 0.88]			0.143			
Cigarettes	43 (1.2)	-1.05	[-2.63, 0.53]	0.194	-0.16	[-1.58, 1.26]			0.826		-1.5		[-2.83, -0.18]		**0.026**		-0.15	[-1.44, 1.14]			0.819			
Vaping	247 (6.9)	0.2	[-0.51, 0.92]	0.582	1	[0.36, 1.65]			**0.002**		0.19		[-0.42, 0.80]		0.536		0.21	[-0.38, 0.80]			0.493			
**Emotional Eating**																								
Eating junk food	1034 (28.9)	0.82	[0.45, 1.19]	**< 0.0001**	0.73	[0.39, 1.07]			**< 0.0001**		-0.51		[-0.82, -0.19]		**0.002**		0.81	[0.50, 1.12]			**< 0.0001**			
**Passive Screen**																								
Video games, TV/movies, internet/social media	2724 (76.2)	0.16	[-0.24, 0.55]	0.439	-0.04	[-0.39, 0.32]			0.837		-0.43		[-0.76, -0.10]		**0.011**		0.02	[-0.30, 0.35]			0.882			
**Hobbies and the Arts**																								
Reading, writing, music, or arts & crafts	1747 (48.8)	0.14	[-0.21, 0.49]	0.428	0.16	[-0.15, 0.48]			0.314		-0.11		[-0.41, 0.19]		0.472		0.26	[-0.02, 0.55]			0.072			
Learning something new	976 (27.3)	-0.01	[-0.39, 0.36]	0.947	0.17	[-0.17, 0.51]			0.328		0.45		[0.13, 0.77]		**0.005**		-0.09	[-0.40, 0.21]			0.547			
Cooking/baking	1762 (49.3)	-0.15	[-0.49, 0.20]	0.407	-0.07	[-0.38, 0.24]			0.654		-0.05		[-0.34, 0.25]		0.754		-0.05	[-0.33, 0.24]			0.753			
Spending time with pet	1527 (42.7)	0	[-0.32, 0.33]	0.986	0.02	[-0.27, 0.32]			0.879		-0.06		[-0.33, 0.22]		0.694		0.2	[-0.07, 0.47]			0.145			
**Caring for Others**																								
Trying to help others	825 (23.1)	0.24	[-0.15, 0.63]	0.226	0.38	[0.02, 0.73]			**0.036**		0.83		[0.50, 1.16]		**< 0.0001**		-0.12	[-0.44, 0.19]			0.446			
**Spirituality and Mindfulness**																								
Meditating or praying	364 (10.2)	0.45	[-0.09, 0.98]	0.104	0.47	[-0.01, 0.96]			0.057		0.28		[-0.18, 0.73]		0.232		-0.25	[-0.70, 0.19]			0.261			
**Structured Lifestyle Behaviours**																								
Getting exercise	2332 (65.2)	-0.22	[-0.58, 0.13]	0.216	-0.04	[-0.36, 0.28]			0.793		0.33		[0.03, 0.63]		**0.032**		0.07	[-0.22, 0.37]			0.612			
Keeping to a regular schedule	1364 (38.1)	-0.85	[-1.19, -0.51]	**< 0.0001**	-0.36	[-0.67, -0.06]			**0.020**		0.48		[0.2, 0.77]		**0.001**		-0.36	[-0.64, -0.08]			**0.011**			
Studying or schoolwork	2540 (71.0)	-0.2	[-0.59, 0.18]	0.300	-0.18	[-0.53, 0.17]			0.306		0.34		[0.01, 0.67]		**0.041**		-0.19	[-0.50, 0.13]			0.238			
**Formal Supports**																								
Mental health professionals	120 (3.4)	2.63	[1.76, 3.51]	**< 0.0001**	2.15	[1.36, 2.93]			**< 0.0001**		-1.34		[-2.08, -0.61]		**0.000**		1.74	[1.03, 2.45]			**< 0.0001**			
**Time Alone**																								
Spending time alone	2080 (58.1)	1.75	[1.41, 2.09]	**< 0.0001**	0.66	[0.35, 0.96]			**< 0.0001**		-1.41		[-1.69, -1.12]		**< 0.0001**		0.87	[0.60, 1.15]			**< 0.0001**			

* A higher score for anxiety, depression, and emotional regulation indicates more symptoms/dysregulation. A higher score for psychosocial well-being represents greater flourishing. Depression: Model adjusted for grade, sex, province, and baseline CESD score. Anxiety: Model adjusted for grade, sex, province, and baseline GAD7 score. Psychosocial well-being: Model adjusted for grade, sex, province, spending money, household size, and baseline FS score. Emotion regulation: Model adjusted for grade, sex, province, spending money, household size, and baseline DERS score. CI: confidence interval. Significant p-values (p < 0.05) are bolded*

### Depression

Students who reported spending time with family (b = -1.95, p < 0.0001) and keeping to a regular schedule (b = -0.85, p < 0.0001) had significantly lower mean increase in depression scores compared to those who did not engage in those coping behaviours. Students who reported coping by drinking alcohol (b = 1.01, p = 0.001), eating junk food (b = 0.82, p < 0.0001), connecting with a mental health professional (b = 2.63, p < 0.0001), and spending time alone (b = 1.75, p < 0.0001) had significantly higher mean increase in depression than those that did not.

### Anxiety

Students who reported spending time with family (b = -1.22, p < 0.0001), using cannabis (b = -1.02, p = 0.012), and keeping a regular schedule (b = -0.36, p = 0.020) reported a significantly lower mean increase in anxiety compared to those that did not engage in these coping behaviours. However, drinking alcohol (b = 0.81, p = 0.004), vaping (b = 1.00, p = 0.002), eating junk food (b = 0.73, p < 0.0001), and trying to help others (b = 0.38, p = 0.036), connecting with a mental health professional (b = 2.15, p < 0.0001), and spending time alone (b = 0.66, p < 0.0001) were associated with a significantly greater mean increase in anxiety scores.

### Psychosocial well-being

Students who reported staying connected with friends online (b = 1.22, p < 0.001), meeting up with friends outside (b = 0.54, p = 0.000), spending time with family (b = 1.52, p < 0.0001), learning something new (b = 0.45, b = 0.005), trying to help others (b = 0.83, p < 0.0001), getting exercise (b = 0.33, p = 0.032), keeping a regular schedule (b = 0.48, p = 0.001), and studying/working on schoolwork (b = 0.34, p < 0.041) exhibited a smaller decrease (less detrimental) in mean FS scores compared to those that did not engage in these coping behaviours. In contrast, drinking alcohol (b = -0.71, p = 0.007), smoking cigarettes (b = -1.5, p = 0.026), eating junk food (b = -0.51, 0.002), playing video games, watching TV or movies, surfing the internet/social media (b = -0.43, p = 0.011), connecting with mental health professionals (b = -1.34, p = 0.000), and spending time alone (b = -1.41, p < 0.0001) were associated with a greater mean decrease in flourishing score than those who did not engage in these behaviours.

### Difficulties in emotion regulation

Students who reported staying connected with friends online (b = -0.45, p = 0.006), spending time with family (b = -1.09, p < 0.0001), and keeping a regular schedule (b = -0.36, p = 0.011) had significantly smaller increase in mean emotion regulation score compared to those that did not engage in these coping behaviours. Students who reported eating junk food (b = 0.81, p < 0.0001), connecting with a mental health professional (b = 1.74, p < 0.0001), and spending time alone (b = 0.87, p < 0.0001) had a significantly greater increase in emotion regulation score compared to students who did not engage in these coping behaviours.

### Gender and coping behaviour interactions

See Tables 3, 4, 5 and 6 for results for least squares means of gender and coping behaviour interactions where main effects for coping variables were found. For females, spending time with family and friends was more beneficial for anxiety, depression, and emotion regulation compared to males. Coping with junk food had a higher detrimental effect on depression, anxiety, and emotion regulation for females compared to males. For females compared to males, learning something new had a detrimental effect on psychosocial well-being while spending time alone was more beneficial for psychosocial well-being.

**Table 3:** Least Squares Means of Gender and Coping Behaviour Interactions for Change in Depression Symptoms Among Adolescents (n = 3492)

Least Squares Means of Interactions for Change in Depression	Females			Males	
	Mean	95% CI	Mean		95% CI
Spending time with my family (e.g., playing games, eating meals together, hanging out)					
Yes	2.04	[0.97, 3.11]*	-0.01		[-1.32, 1.29]
No	4.29	[3.21, 5.37]	1.43		[0.11, 2.75]
**Using cannabis/marijuana**					
Yes	3.74	[2.59, 4.88]	0.93		[-0.54, 2.39]
No	2.59	[1.51, 3.67]	0.49		[-0.80, 1.78]
**Eating junk food**					
Yes	3.73	[2.65, 4.81]**	0.80		[-0.53, 2.14]
No	2.60	[1.53, 3.67]	0.61		[-0.70, 1.92]
**Keeping to a regular schedule (e.g., waking up, eating meals, and going to bed around the same time as usual)**					
Yes	2.64	[1.56, 3.73]	0.43		[-0.90, 1.76]
No	3.68	[2.62, 4.75]	0.98		[-0.32, 2.28]
**Connecting with mental health professionals**					
Yes	4.58	[3.23, 5.93]	1.60		[-0.40, 3.61]
No	1.75	[0.80, 2.70]	-0.19		[-1.19, 0.81]
**Spending time alone**					
Yes	4.04	[2.99, 5.10]	1.58		[0.29, 2.86]
No	2.28	[1.20, 3.37]	-0.16		[-1.50, 1.17]

CI: confidence interval; * indicating engaging in the coping behaviour was more beneficial and ** indicating it was more detrimental for females relative to males

**Table 4:** Least Squares Means of Gender and Coping Behaviour Interactions for Change in Anxiety Symptoms Among Adolescents (n = 3531)

Least Squares Means of Interactions for Change in Anxiety	Females			Males	
	Mean	95% CI	Mean		95% CI
Spending time with my family (e.g., playing games, eating meals together, hanging out)					
Yes	2.28	[1.30, 3.26] *	0.86		[-0.38, 2.10]
No	3.90	[2.92, 4.88]	1.41		[0.16, 2.65]
**Using cannabis/marijuana**					
Yes	2.33	[1.21, 3.45]	1.13		[-0.43, 2.69]
No	3.85	[2.84, 4.87	1.13		[-0.09, 2.35]
**Drinking alcohol**					
Yes	3.48	[2.45, 4.52]	1.43		[0.10, 2.76]
No	2.70	[1.70, 3.70]	0.83		[-0.45, 2.12]
**Vaping**					
Yes	3.78	[2.72, 4.84]	1.25		[-0.14, 2.65]
No	2.40	[1.38, 3.42]	1.01		[-0.27, 2.28]
**Eating junk food**					
Yes	3.58	[2.59, 4.56]**	1.23		[-0.02, 2.48]
No	2.60	[1.63, 3.58]	1.03		[-0.22, 2.28]
**Trying to help others**					
Yes	3.24	[2.24, 4.24]	1.42		[0.14, 2.70]
No	2.94	[1.98, 3.91]	0.84		[-0.38, 2.07]
**Keeping to a regular schedule (e.g., waking up, eating meals, and going to bed around the same time as usual)**					
Yes	2.88	[1.89, 3.87]	1.01		[-0.24, 2.26]
No	3.30	[2.33, 4.20]	1.25		[0.02, 2.49]
**Connecting with mental health professionals**					
Yes	4.28	[3.06, 5.50]	1.76		[-0.06, 3.58]
No	1.90	[1.03, 2.78]	0.50		[-0.50, 1.50]
**Spending time alone**					
Yes	3.35	[2.38, 4.31]	1.62		[0.40, 2.84]
No	2.84	[1.84, 3.83]	0.64		[-0.62, 1.91]

CI: confidence interval; * indicating engaging in the coping behaviour was more beneficial and ** indicating it was more detrimental for females relative to males

**Table 5:** Least Squares Means of Gender and Coping Behaviour Interactions for Change in Emotion Dysregulation Among Adolescents (n = 3509)

Least Squares Means of Interactions for Change in Emotion Regulation	Females		Males		
	Mean	95% CI	Mean	95% CI	
Staying connected with friends online					
Yes	1.61	[0.76, 2.46]*	0.65	[-0.35, 1.64]	
No	2.42	[1.54, 3.31]	0.43	[-0.64, 1.51]	
**Spending time with my family (e.g., playing games, eating meals together, hanging out)**					
Yes	1.42	[0.56, 2.28]*	0.09	[-0.93, 1.11]	
**No**	2.62	[1.75, 3.48]	0.99	[-0.04, 2.02]	
**Eating junk food**					
Yes	2.55	[1.68, 3.42]**	0.66	[-0.39, 1.71]	
No	1.48	[0.63, 2.33]	0.42	[-0.59, 1.44]	
**Keeping to a regular schedule (e.g., waking up, eating meals, and going to bed around the same time as usual)**					
Yes	1.76	[0.89, 2.63]	0.52	[-0.52, 1.56]	
No	2.27	[1.42, 3.12]	0.56	[-0.45, 1.58]	
**Connecting with mental health professionals**					
Yes	2.88	[1.80, 3.97]	1.24	[-0.34, 2.81]	
No	1.15	[0.39, 1.91]	-0.15	[-0.95, 0.64]	
**Spending time alone**					
Yes	2.42	[1.57, 3.27]	1.04	[0.04, 2.05]	
No	1.61	[0.74, 2.48	0.04	[-1.01, 1.08]	

CI: confidence interval; * indicating engaging in the coping behaviour was more beneficial and ** indicating it was more detrimental for females relative to males

**Table 6:** Least Squares Means of Gender and Coping Behaviour Interactions for Change in Flourishing Among Adolescents (n = 3556)

Least Squares Means of Interactions for Change in Flourishing	Females			Males	
	Mean	95% CI	Mean		95% CI
Staying connected with friends online					
Yes	-1.72	[-2.74, -0.71]	0.18		[-1.01, 1.36]
No	-2.96	[-4.01, -1.91]	-0.80		[-2.06, 0.45]
**Meeting up with my friends outside**					
Yes	-2.12	[-3.15, -1.09]	0.06		[-1.16, 1.27]
No	-2.56	[-3.58, -1.54]	-0.69		[-1.91, 0.54]
**Spending time with my family (e.g., playing games, eating meals together, hanging out)**					
Yes	-1.59	[-2.62, -0.57]	0.45		[-0.77, 1.67]
No	-3.09	[-4.11, -2.06]	-1.08		[-2.30, 0.14]
**Drinking alcohol**					
Yes	-2.80	[-3.87, -1.74]	-0.47		[-1.75, 0.81]
No	-1.88	[-2.93, -0.83]	-0.16		[-1.43, 1.11]
**Smoking cigarettes**					
Yes	-3.52	[-5.26, -1.78]	-0.76		[-2.69, 1.17]
No	-1.16	[-1.88, -0.43]	0.13		[-0.92, 1.18]
**Eating junk food**					
Yes	-2.58	[-3.61, -1.55]	-0.58		[-1.81, 0.65]
No	-2.10	[-3.12, -1.08]	-0.05		[-1.26, 1.16]
**Playing video games, watching TV or movies, surfing the internet/social media**					
Yes	-2.50	[-3.52, -1.48]	-0.80		[-1.99, 0.39]
No	-2.18	[-3.22, -1.15]	0.17		[-1.11, 1.46]
**Learning something new (painting, playing a musical instrument, a new language)**					
Yes	-2.24	[-3.28, -1.20]**	0.18		[-1.07, 1.43]
No	-2.44	[-3.46, -1.42]	-0.81		[-2.01, 0.39]
**Trying to help others**					
Yes	-1.87	[-2.91, -0.82]	-0.05		[-1.31, 1.20]
No	-2.81	[-3.83, -1.80]	-0.57		[-1.77, 0.63]
**Getting exercise (e.g., getting outside to go for a walk or bike ride, working out at home)**					
Yes	-2.25	[-3.27, -1.23]	-0.03		[-1.24, 1.19]
No	-2.44	[-3.47, -1.40]	-0.60		[-1.82, 0.62]
**Keeping to a regular schedule (e.g., waking up, eating meals, and going to bed around the same time as usual)**					
Yes	-2.08	[-3.12, -1.05]	-0.10		[-1.33, 1.13]
No	-2.60	[-3.62, -1.58]	-0.53		[-1.74, 0.68]
**Studying or working on schoolwork**					
Yes	-2.07	[-3.09, -1.05]	-0.24		[-1.45, 0.97]
No	-2.61	[-3.66, -1.57]	-0.39		[-1.61, 0.84]
**Connecting with mental health professionals**					
Yes	-3.09	[-4.33, -1.86]	-0.54		[-2.24, 1.17]
No	-1.59	[-2.53, -0.65]	-0.09		[-1.16, 0.97]
**Spending time alone**					
Yes	-2.92	[-3.93, -1.91]*	-1.24		[-2.43, -0.04]
No	-1.76	[-2.80, -0.72]	0.61		[-0.62, 1.84]

CI: confidence interval; * indicating engaging in the coping behaviour was more beneficial and ** indicating it was more detrimental for females relative to males

## Discussion

The most common COVID-19 coping behaviours reported by adolescents included staying connected with friends online, spending time with family, engaging in screen media use, studying or working on schoolwork, and getting exercise. Few students reported using substances or connecting with mental health professionals to cope with the pandemic. In line with our findings, a UK study examining the well-being and coping experiences of 16–19 years olds in the first COVID-19 lockdown found that adolescents reported the importance of togetherness, developing a routine or structure to keep busy, and use of technology to stay connected with people outside their household during the lockdown [49].

The use of positive coping mechanisms during the early pandemic period (e.g., schedule, family, friends) was associated with less detrimental within-individual changes across the mental health outcomes examined, whereas negative coping mechanisms (e.g., time alone, eating junk food) were consistently associated with more adverse mental health changes from before to during the early lockdown. Additionally, spending time with family and friends was more beneficial for females compared to males for anxiety, depression, and emotion regulation, while coping with junk food had a higher detrimental effect on females across all measures. This study provides prospective evidence to support the need to ensure adolescents have the resources, support, and encouragement to engage in positive coping behaviours for the prevention of adverse mental health changes. Targeted interventions appear warranted for populations who are more likely to be isolated alone and less likely to spend time with family or online with friends and to retain a routine/structure.

### Consistent positive coping behaviours

Keeping a regular schedule, such as by waking up, eating meals, and going to bed around the same time, was associated with less detrimental changes in all mental health outcomes at the start of the pandemic. Consistent bed and wake times are in line with the recommendations from the Canadian 24-Hour Movement Guidelines for practicing healthy sleep hygiene [50]. A qualitative study found that routines helped improve stability and continuity in family environments, especially in times of stress as well as facilitating feelings of control and protecting well-being [51]. Keeping with a routine during school closures may help with a sense of normalcy; however, this could be difficult due to disruption of regular schedules (e.g., remote learning, no dedicated space to work/learn, etc.). Interventions to support adolescents in planning and maintaining a regular schedule regardless of school format may prove valuable.

Over half of students reporting coping by spending time with family, which was associated with less deterioration in mental health. Staying connected with friends online was the most commonly reported coping behaviour and was associated with more positive changes in psychosocial well-being and emotion regulation, but not with depression or anxiety. Perceiving the presence of support appears to deter use of more maladaptive coping strategies such as withdrawal and avoidance [52, 53] and feelings of social connectedness have shown to be protective against poor mental health in adolescents during the pandemic [54].

It is crucial to acknowledge that not all students have the privilege of utilizing positive coping behaviours. For instance, youth in rural, remote, and low-income households are less likely to have access to devices and highspeed internet [55]. Furthermore, coping with family time may be contingent on having a happy home life, which has been consistently found to be a strong predictor of youth mental health outside of the pandemic [56, 57]. Increased conflict with parents during the pandemic was associated with increased mental health problems [54]. These household stresses may stem from financial insecurity (e.g., unemployment, inadequate financial relief packages), confinement-related stress (e.g., crowding) [58], a toxic or dysfunctional home environment [59], or having parents who were essential or frontline workers (e.g., lack of support at home) [60, 61]. Further research is needed to confirm associations found in this study while controlling for these potential confounders.

### Consistent negative coping behaviours

Four coping behaviours – connecting with a mental health professional, spending time alone, eating junk food, and using substances (i.e. alcohol) – were consistently associated with more negative mental health changes. However, it is important to recognize in interpreting our results that we are unable to determine directionality. Adolescents who experienced more adverse mental health changes may have reached out to mental health supports. More likely, adolescents reporting coping this way during COVID-19 pandemic may have already been seeing a mental health professional before the onset of the pandemic. Post-hoc analyses indicated poorer pre-COVID-19 mental health scores across the four scales in students that indicated connecting with a mental health professional during the pandemic compared to their peers that did not report coping in this way (Table S1). Previous evidence indicates that individuals with pre-existing mental health problems and illness were more likely to be adversely impacted by the pandemic [62]. Connecting with a mental health professional was one of the least commonly reported coping behaviours in this study. It is plausible that some adolescents had difficulty accessing appropriate care and services during this time [10, 13]. This may arise from the lack of autonomy as a barrier for younger adolescents seeking out professional support, not knowing where to get help, and excessive wait times and costs for seeing a mental health professional, which have been further exacerbated during the pandemic [13].

Spending time alone as a coping strategy was associated with more negative changes in all four mental health outcomes. Concerningly, spending time alone was reported by over half of adolescents, about the same proportion that reported spending time with family to cope. Results support the importance of socialization for adolescents [63, 64]. Previous research has found that low social support is associated with higher incidence of anxiety and depressive symptoms among adolescents [65, 66]. It is unclear from our analysis whether spending time alone was a maladaptive coping strategy and/or if social withdrawal was a sign of distress [67]. Alternatively, adolescents who reported spending time alone to cope may not have had access to positive peer/family relationships. Other research examining students’ feelings of social connectedness at school have found that those students reporting poor social connectedness (e.g., no one to talk to, trust, depend on, etc.) were two to three times more likely to experience depressive symptoms compared to students who reported having positive relationships [68].

Eating junk food as a coping strategy was reported by about 29% of adolescents and may stem from the environment (e.g., at home during online schooling), accessibility to food, and/or boredom or lack of routine during the pandemic. Consuming junk food may be a response to emotional distress and negative emotions [69] and perceived stress may contribute to greater consumption of junk food and disinhibited eating/binge eating [70]. Similarly, alcohol was a maladaptive coping behaviour for three of the four mental health outcomes. Almost one tenth of adolescents reported using alcohol to cope with the pandemic. Although adolescent alcohol use is typically a social activity, recent evidence found that frequency of alcohol use increased in adolescents [71] and more generally [72]. Dumas et al. (2020) found that while the majority of substance use (49.3%) was solitary, many adolescents adapted to using substances with peers via technology (31.6%) and face-to-face (23.6%). Additionally, other research points to alcohol use as a way to contend with traumatic events [73] or stress [74]. It may be prudent for efforts to focus on educating/delivering information to adolescents about ways to mitigate maladaptive coping mechanisms.

### Inconsistent coping behaviours

Seven coping behaviours were inconsistent in their associations with changes across the mental health outcomes: studying/working on schoolwork, trying to help others, getting exercise, passive screen use, and other substance use (vaping, smoking cigarettes, cannabis). Students that reported studying or working on schoolwork as a coping behaviour had less adverse changes in their psychosocial well-being. Canadian schools varied in their response during the early pandemic lockdown, with some sending work home or engaging online, and many essentially having an extended summer holiday. Continuing to complete schoolwork or study may have provided students a sense of routine and normalcy, allowing a somewhat regular schedule.

Students who reported trying to help others as a coping strategy showed a negative change in reported anxiety and a more positive change psychosocial wellbeing. The former may have resulted from the stress of having to take care of someone (e.g., immunocompromised family member, a younger sibling) whether voluntary or out of necessity, and perhaps not caring for oneself in the process. In contrast, trying to help others (e.g., volunteering) may contribute to feelings of being ‘a part of something’, sense of community/connection, or life satisfaction [75, 76]. Results align with previous evidence of links between mental health and prosocial behaviour in adolescents [77].

Unsurprisingly, students that reported using exercise as a coping behaviour had better change in psychosocial wellbeing, which is in line with literature on benefits of physical activity for mental and physical health [78–80]. This item also may reflect the positive influence of getting outside during this period [81]. Future interventions may want to focus on promoting physical activity and routine, and helping adolescents establish a sense of community and social connection, while also focusing on self care and self-compassion [82].

Coping behaviours involving substance use, vaping and smoking cigarettes, were associated with worsening anxiety and poorer psychosocial well-being respectively. Conversely, cannabis was associated with more positive changes in anxiety. While some research supports cannabinoid therapies as potential treatments in managing anxiety [83], research in adolescents has typically found cannabis use to be associated with increases in depression and anxiety symptoms [84] and to predict adverse mental health changes over time [85].

Unlike connecting with friends online, less social or more passive forms of screen use (e.g., video games, watching TV/movies, surfing the internet) were associated with more negative psychosocial well-being changes. Results are in line with a scoping review that found high levels of screen time associated with unfavourable psychological outcomes (e.g., mental health, cognitive functioning) [86] and specifically, psychological well-being [87]. As this measure grouped different forms of screen use together, we are unable to comment on whether they differ. With pandemic public health restrictions that limited places where adolescents could go, passive screen time may have helped fill the time. Specifically, social media use may provide avenues for social connection while at the same time potentially creating a source of isolation and exposure to negative content such as cyberbullying [88]. Conversely, more time spent on screens at home may deter more positive coping behaviours, such as physical activity, keeping a routine and consistent bedtimes, and spending time with family.

The COVID-19 pandemic created a unique phenomenon, in that it provides uncertainty, life threatening conditions, as well as extended exposure to stress-inducing information [5, 16]. These factors create extended stressors that adolescents must address through various coping strategies. However, the pandemic also has removed go-to coping strategies that adolescents may have utilized pre-pandemic (e.g., face-to-face interactions with peers, consistent routines, mental health support in the school setting) and research finds that depending on their development stage, children and adolescents responded differently to pandemic stresses [89]. It is of utmost importance to support all adolescents, regardless of gender, socioeconomic status, and location, with the tools and coping strategies that will help promote positive mental health outcomes.

There are challenges in determining what is ‘clinically meaningful’ within the context of this study. Minimal clinically important differences do not account for multiple simultaneous behaviours. That is, the effect size of an individual coping behaviour may not reach a minimal clinically importance difference threshold, but the adoption of multiple positive coping behaviours may add up to a larger meaningful difference, since they are not mutually exclusive. This study modeled the data by including all coping behaviours as predictors simultaneously; individuals adopting multiple, good coping behaviours and avoiding deleterious ones, can potentially add up to larger differences since these coping strategies are not mutually exclusive. Also, the simultaneous adoption of deleterious coping behaviours needs to be considered. Future analyses would need to consider additive effects of various combinations of coping behaviours to determine clinical meaningfulness.

### Strengths & limitations

The strengths of this study included a large prospective sample of Canadian adolescents with linked data from before to the early pandemic lockdown. Additionally, the study used well-validated mental health measures and examination of both psychopathology and mental well-being. Limitations of this study are the lack of information on the type of coping strategies students used prior to the pandemic and the frequency and intensity of coping behaviours during the pandemic, given the checklist measure used. Also, the one-year interval between data collections may not be sensitive enough to detect some shorter-term changes. Further research is needed to examine how coping behaviours and mental health changed over the prolonged pandemic response. Furthermore, as COMPASS was not designed to be representative, the prevalence of coping behaviours and mental health scores may not be generalizable to all Canadian adolescents; however, relationships should be generalizable when there is sufficient heterogeneity in exposures and outcomes. With the shift to an online COMPASS survey, we experienced lower online response rates, which may bias the results due to self-selection. Students experiencing poorer mental health during the pandemic and engaging in more maladaptive coping behaviour may have been less likely to participate. Additionally, the shift from school-based paper-and-pencil questionnaires to an online survey format may have influenced reporting. Furthermore, self-report data is prone to recall error and social desirability bias. However, COMPASS uses passive consent protocols which are shown to better reach students at risk of depression [90], and does not require student names, helping to preserve perceptions of anonymity for honest reporting.

## Conclusion

Overall, this study adds to evidence of the importance of social support for adolescent mental health, given the positive results for spending time with family or online with friends, and negative results for spending time alone. Keeping and maintaining a routine or structure also emerged as positive coping strategy. Future research and interventions targeting coping behaviours should consider focusing on how to make these coping strategies equitable across secondary school students, as well as examining the long-term impacts of the pandemic on mental health outcomes and consequent coping behaviours.

## Electronic supplementary material

Below is the link to the electronic supplementary material.

Supplementary Material 1

## Data Availability

COMPASS study data is available upon request through completion and approval of an online form: https://uwaterloo.ca/compass-system/information-researchers/data-usage-application The datasets used during the current study are available from the corresponding author on reasonable request.
