# Supplementary material for: Stronger together: Coping behaviours and mental health changes of Canadian adolescents in early phases of the COVID-19 pandemic
Source: BMC Public Health. 2023 Feb 13;23:319. doi: 10.1186/s12889-023-15249-y (PMC9924880; doi:10.1186/s12889-023-15249-y)
Supplement: Supplementary file 1 — Supplementary Material 1 [file 12889_2023_15249_MOESM1_ESM.doc]

**Supplementary Table S1. Post hoc analysis examining association between connecting with mental health professionals to cope and baseline mental health scores**

| **MH Scale Score at Baseline (Year 7):** | **Depression** | | | **Anxiety** | | **Psychosocial  well-being** | | | | **Emotion Dysregulation** | |
| --- | --- | --- | --- | --- | --- | --- | --- | --- | --- | --- | --- |
| **Connecting with mental health professionals:** | **No** | **Yes** | **No** | | **Yes** | | **No** | **Yes** | **No** | | **Yes** |
| n | 3396 | 117 | 3420 | | 119 | | 3438 | 119 | 3404 | | 118 |
| Mean | 8.2 | 12.2 | 5.8 | | 8.9 | | 32.9 | 30.4 | 13.9 | | 15.7 |
| Std Dev | 5.9 | 7.4 | 5.1 | | 5.6 | | 5.4 | 6.5 | 4.7 | | 5.0 |
| T-test p-value | <.0001 | | <.0001 | | | | <.0001 | | <.0001 | | |
